# Economic growth and electricity consumption: Fresh evidence of panel data for LAC

**DOI:** 10.1016/j.heliyon.2024.e33521

**Published:** 2024-06-25

**Authors:** Ciro Eduardo Bazán Navarro, Juan Daniel Morocho Ruiz, Juan Francisco Castillo Alvarado

**Affiliations:** a Center for Economic Research and Sectoral and Social Policies, Economics Programs, Faculty of Business Sciences, Universidad San Ignacio de Loyola, Lima, 15024, Peru; b Graduated from the economics career of the National University of Piura (UNP), Piura, Peru; c Bachellor from the economics career of the National University of Piura (UNP), Piura, Peru

**Keywords:** Electricity consumption, Economic growth, Panel data, Latin America, Caribbean

## Abstract

This study reexamines the causal nexus among electricity consumption (EC) and economic growth (EG) for a panel of 31 countries in Latin America and the Caribbean between 1980 and 2021. We find that there are statistically significant feedback impacts among the research variables in the long run. A 1 percent augment in EC raises EG by 0.5 percent and a 1 percent augment in EG produces a 1.54 percent increase in EC which reflects the nature of the latter as a luxury good and implies a tradeoff between economy and environment, since although greater electrical infrastructure drives EG, the latter also increases the EC whose use in a non-responsible manner could lead to environmental degradation through higher CO_2_ emissions. Therefore, the main policy implication is that, it is necessary to promote EG based on infrastructure focused on sustainable development, ensuring the well-being of present and future generations.

## Abbreviations

Causality relationship: (CR)
Conservation policies: (CP)
Direction of Causality: (DOC)
Dynamic ordinary least square: (DOLS)
Electricity consumption: (EC)
Electricity consumption per capita: (ECpc)
Economic growth: (EG)
Error correction: (EC)
Empirical evidence: (EE)
Fixed capital formation: (FCF)
Foreign direct investment: (FDI)
Fully modified ordinary least square: (FMOLS)
Granger causality: (GC)
Greenhouse gas: (GHG)
Gross Domestic Product per capita: (GDPpc)
Growth rate: (GR)
Latin America and the Caribbean: (LAC)
Labor force: (LF)
Long run: (LR)
Null hypothesis: (H0)
Organization for Economic Co-operation and Development: (OECD)
Panel cointegration: (PC)
Panel data: (PD)
Panel Granger causality: (PGC)
Pooled mean group estimator: (PMGE)
Short run: (SR)
Statistical significance: (SS)
Statistically significant: (SST)
Sustainable development: (SD)
Unit root: (UR)
Unit root test: (URT)

## 1 Introduction

LAC appears as one of the world regions with the most important growth in the ECpc in the last 30 years. Between 1980 and 2021, the ECpc has grown 169 %, with an average annual GR of 2,5 %, only behind Asia (average annual GR of 4,8 %). Furthermore, although GDPpc in the region has increased in 38 %, the average annual GR has been only of 0,8 %, surpassing only Africa (average annual GR of 0.7 %). As a result, the rate between total EC and GDP, an approximate measure of how much electric energy is required to generate one unit of GDP, has increased in 95 % during that period, becoming the region with the major increase in this rate. These statistics are particularly relevant considering that, according to Ref. [1], the 55 % of the GHG emissions in LAC are attributed to the energy sector.

**Fig. 1:**
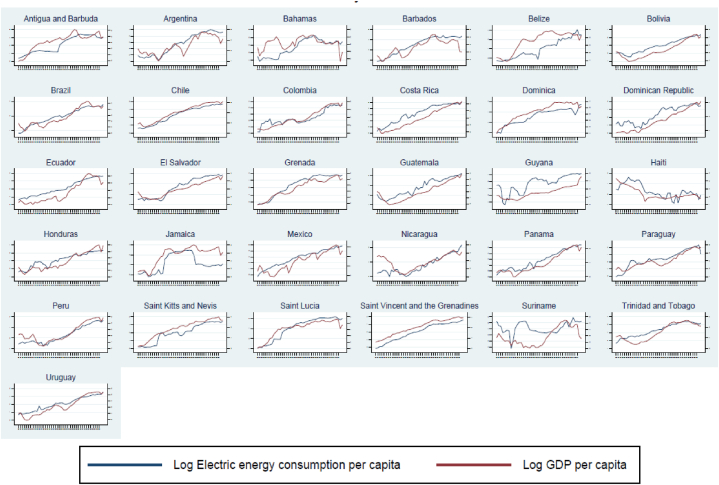
Developed by authors. **Source:** U.S. Energy Information Administration and World Bank. Relationship between ECpc (kWh) and GDPpc (USD, 2015 = 100) by country, 1980–2021. Panel 1: Antigua and Barbuda, Panel 2: Argentina, Panel 3: Bahamas, Panel 4: Barbados, Panel 5: Belize, Panel 6: Bolivia, Panel 7: Brazil, Panel 8: Chile, Panel 9: Colombia, Panel 10: Costa Rica, Panel 11: Dominica, Panel 12: Dominican Republic, Panel 13: Ecuador, Panel 14: El Salvador, Panel 15: Grenada, Panel 16: Guatemala, Panel 17: Guyana, Panel 18: Haiti, Panel 19: Honduras, Panel 20: Jamaica, Panel 21: Mexico, Panel 22: Nicaragua, Panel 23: Panama, Panel 24: Paraguay, Panel 25: Peru, Panel 26: Saint Kitts and Nevis, Panel 27: Saint Lucia, Panel 28: Saint Vincent and the Grenadines, Panel 29: Suriname, Panel 30: Trinidad and Tobago, Panel 31: Uruguay.

According to Fig. 1, a first snapshot at a descriptive and statistical level suggests a potential positive association between the ECpc and the EG (measured by the GDPpc) for the economies of the LAC region. These economies, with the exception of 2020 (coronavirus pandemic), have generally shown positive performance in GDPpc levels. Similar behavior has also been evident for the region's ECpc. Likewise, during the period 1980–2021 the correlation between both variables has been 85.87 %, positive and SST at the 5 % level. However, although both variables show a potential positive and SST association, it is relevant to identify the DOC between these variables.

Thus, it is necessary to know if the EG of LAC region has been promoted by the EC or vice versa, as well as to examine the possibility of a bidirectional relationship or absence of any relationship between EG and EC. The DOC existing between both variables has important implications in the design and effective execution of energy and environmental policies [2]. In particular, if it is corroborated that the EG is driven by the EC (Growth hypothesis, GH), the relevance of this last variable as a production factor for the economy is revealed [3]. This in turn leads to a country (economy) being called dependent on electrical energy. However, the validation of this DOC also could generate concern since the occurrence of negative shocks in the energy supply would have a negative effect on the EG Ref. [4].

In contrast, if the DOC goes from EG to EC, this would show evidence of the Conservation hypothesis (CH) of electrical energy, which implies that EG drives EC which in turn leads to positive impacts mainly in education, health, labor, and productivity [5]. However, the verification of this CR could create an unfavorable scenario if the EG led to excessive growth in the demand for electrical energy, since this could increase environmental degradation through greater CO_2_ emissions [6]. Likewise, the DOC between EC and EG can be bidirectional (Feedback hypothesis, FH) as well as implies the absence of any CR (Neutrality hypothesis, NH). Under the verification of FH, the implementation of energy CP which reduce EC will reduce the EG and this last reduction will in turn reduce the EC ([3,7]). Under the validation of NH, it is stipulated that the energy CP whose purpose is to reduce the EC will not affect to EG Ref. [8]. For more details on the theoretical hypotheses about the CR between EC and EG, see Refs. [4,9].

In this sense, the analysis of the nexus among EG and EC in LAC is of vital interest. First, given the potential association between both variables under study. Secondly, it must be considered that both variables reflect a trade-off between economy and environment, given a context of SD Goals (SDGs) within which the aim is to promote access to affordable, reliable and sustainable energy as well as to take action to combat the climate change through the management of GHG emissions. Thirdly, the main reason for considering LAC as a geographical area under study is based on the generation of new EE on the nexus between GE and CE for this region, since the vast majority of empirical studies that address this relationship only analyzes a group of countries in the world without considering LAC as a whole. In particular, in the aforementioned studies, only a subsample of countries of LAC region is examined, and the results associated with said region or their implications on the issue of the existing balance between economy and environment are not specifically addressed. Therefore, this research focuses its analysis on the existing tradeoff between economy and environment in LAC, raising its main conclusions and policy implications with emphasis on a harmonious balance between the economic and the environmental, which constitutes a key aspect in the SD of the region, and as a basis to guarantee the well-being of present and future generations.

In this framework, the purpose of this paper is to make available fresh evidence on the dynamic nexus between EC and EG for a set of 31 LAC countries between 1980 and 2021. To such aim, we implemented a panel URT, a panel cointegration test, and a PGC test based on the vector EC model (VECM). The main contributions of this study are as follows: First, it uses a recent database to contrast the type of CR between EC and EG for the specific case of LAC. Second, prior research has investigated this topic only considering a sample of all the countries in the region; moreover, a detailed analysis of the results pertaining to this region and to this area of study has not been provided. Finally, the research allows to recognize the importance of the nexus between these variables and its implications related to economy and the environment for the SD of the LAC countries.

After the introduction, an analysis of the previous research on the nexus among variables under study is presented. In Section 2, the methodology used to estimate the nexus among the variables under study is outlined. In Section 3, the outcomes are displayed. In Section 4, the outcomes are reviewed. Lastly, in Section 5, the research conclusions are outlined.

### 1.1 Literature review

In this point, we examine the papers that use techniques of PD to analyze GC and cointegration relationships between GDP and EC for sets of countries, geographic areas, provinces, or states of a given country. We also consider empirical studies that analyzes causality and cointegration between EG and EC in the short and/or longer term, using various control variables (e.g., export/import, energy pricing, CO_2_ emissions, urbanization rate, FDI, FCF) [7,[10], [11], [12]].

#### 1.1.1 Growth hypothesis (GH)

Tiwari et al. [13] examine GC between net state domestic product and ECpc at the state level, between net state domestic product in the agricultural sector (NSDPA) and ECpc in the agricultural sector (PCPCA), and between net state domestic product in the industrial sector (NSDPI) and ECpc in the industrial sector for the 18 largest states in India between 1960/1 and 2014/5. To do so, the authors performed stationarity testing ([[14], [15], [16], [17]]), PD cointegration testing with structural breaks [18] and heterogeneous panel causality [19] using the impulse response function in a panel vector autoregressive (VAR) model. Nazlioglu and Karul's test (see, for example [17]) results provide evidence that all variables are I(1). Moreover, their results provide evidence of cointegration between NSDPA and PCPCA. One of their results shows evidence of causality from PCPCA to NSDPA in the agricultural sector.

Churchill and Ivanovski [8] analyze the linkage between EC and gross state product, along with capital and labor, for a panel of seven Australian states/territories between 1990 and 2015. Their outcomes show that all variables are I(1), and that they are cointegrated. Estimations for the whole panel with the FMOLS, DOLS, and autoregressive distributed lag (ARDL) models show that capital, EC, and labor positively and significantly affect gross state product in the LR. Similarly, for Northern Territory and Queensland, LF and EC have a positive and significant effect in the SR. Regarding causality, the results show an effect on gross state product in the SR. In the LR, the researchers find positive and SST impacts of EC on gross state product in South Australia, Queensland, Tasmania, New South Wales, and Victoria. In the SR, they find a positive and SST effect of EC on gross state product in causalities from EC to gross state product, from gross state product to LF, and from EC to gross FCF. Similarly, bidirectional causalities are found between EC and LF, FCF and gross state product, and LF and gross FCF.

In [20] it is analyzed the link between EC, GDP, FCF, oil price, and population in 210 countries (income level, full panel, OECD and non-OECD level, regional level, renewable energy, and oil imports/exports) for the period 1960–2014. The applied URTs show evidence of series I(1) in the full panel. The authors also use panel cointegration tests, FMOLS, and PGC tests based on VECM. In the SR, their results validate the GH in non–OECD countries, Europe, North Africa, Central Asia, and the Middle East. In addition, the oil price results support a CR from GDP to oil price in high income countries, upper middle–income countries, Europe, OECD countries, Sub–Saharan region, and South Asia, Central Asia, as well as of no CR between GDP and oil price in the Caribbean, Latin America, Middle East, and North Africa region. In the LR, the authors find a significant negative linkage among oil price and GDP in full panel, upper middle-income countries, high income countries, OECD countries, East Asia and Pacific, LAC, and Sub-Saharan Africa region; moreover, there is a significant positive relationship between oil price and GDP in lower middle income, non-OECD, Middle East, North Africa, and South Asian countries. Lastly, there is a significant positive nexus among gross capital formation and GDP in North America, Middle East, Sub-Saharan Africa, North Africa, Europe, North Africa, Central Asia, full panel, and lower and upper middle–income countries.

Apergis and Payne [2] analyze the long- and SR linkages, as well as the CR, between EG and EC for 88 countries grouped into 4 panels as per the World Bank's income classification between 1990 and 2006. The variables used are EC in kWh, real GDP, and measures of real labor and capital. The methodology used is the PC analysis proposed by Ref. [21], and GC with EC is applied. To ascertain the presence of a UR, we use the tests by Refs. [15,22]. For all income levels, excluding the low–income level, the variables are cointegrated. The coefficients obtained with Larsson's method show that the changes in GDP due to changes in EC become smaller as the income level related with each panel increases. As a first result, the EC models show causality from EC to GDP in the SR for the panel of lower middle–income countries. Finally, in the case of the panel of low–income countries, as the variables are not cointegrated, an autoregressive panel is used to determine the CR, and a relationship from EC to GDP is observed.

In [23] it is explored the CR between GDPpc, ECpc, and exports, of a panel of six Middle Eastern countries between 1974 and 2002. The panel URT by Ref. [24] shows that the three variables contain a panel UR. In the SR, the PGC test shows CR running from ECpc to GDPpc and from GDPpc to exports. In the LR, the PGC test confirms causality from exports to ECpc and from exports to GDPpc. The Westerlund test [25] validates the cointegration between GDPpc, ECpc, and exports.

#### 1.1.2 Conservation hypothesis (CH)

Tiwari et al. [13], by means of heterogeneous PD methodologies, also find a CR from net state domestic product to ECpc at both the industrial sector and aggregate state level in the 18 largest states of India between 1960/1 and 2014/5.

Wang et al. [26] analyze CR s between GDPpc, ECpc, and urbanization indicators at national and provincial levels in China using PD for the period 2000–2017. The results of URT by Ref. [15] show that all series are I(1), while the test by Ref. [27] showed that all research variables are cointegrated. In the eastern region, the authors find evidence of causality from GDPpc to ECpc in the SR. Finally, in the central region, a CR is found from GDPpc to ECpc in the LR.

In [10] it is analyzed the causality among EG and ECpc in Organization of the Petroleum Exporting Countries (OPEC) countries from 1980 to 2011. The variables used are EC, real GDPpc, and foreign trade. The methodology used is the PC analysis of Pedroni [28,29] and a FMOLS estimation, as well as GC with error correction. To determine the existence of a UR, they use test proposed by Ref. [30]. As a first result [10], find causality from GDPpc to ECpc in the LR.

In [31], using PD for 160 countries between 1980 and 2010, it is examined the dynamic nexus between ECpc and GDPpc. In the SR, they find causality running from EG to EC, supporting the CH in Pacific, East Asia, Middle East, North Africa, and lower middle–income countries.

Mohammadi and Amin [7] examine dynamics among real GDPpc and EC for 79 countries between 1971 and 2011. They grouped the countries according to EG: low, high, and negative. They find that the series are I(1), based on the URTs ([16,32]). Using the tests by Ref. [33], these authors find cointegration between real GDPpc and EC in high- and low-growth countries but not in countries with negative growth. Similarly, the estimates of the LR elasticity of real GDPpc with regard to EC are SST in countries with positive growth. In addition, the results obtained through EC models suggest a GC from production to energy for countries with negative EG.

Narayan et al. [34], analyze the LR GC between EC and real GDP of seven panels (Western Europe, Africa, Asia, the Middle East, Latin America, G6 and global), which together make up 93 countries between 1980 and 2006. The URT by Ref. [16] shows that the natural logarithm of EC and real GDP are non-stationary in the 7 panels. After applying the test by Refs. [34,35] find that in the Middle East, GDP is a LR driver of EC.

Chen et al. [36] examine the linkage among EC and GDP for 10 industrialized Asian countries using individual country data and PD for the period 1971–2001. The variables used are real GDP and EC. For the individual analysis, the URTs by Refs. [37,38], the Johansen and Juselius [39] cointegration analysis, and the error-corrected GC test are used. For the panel analysis, the methods used are those proposed by Refs. [14,15], and [22], for panel URTs, as well as Pedroni cointegration analysis, and GC with error correction. In the case of individual country data, the Phillips-Perron test is used as a criterion to verify whether the series are non-stationary. Similarly, the Johansen and Juselius cointegration tests (see Ref. [39]) show a cointegration relationship for 7 of the 10 countries (no relationship is found for Malaysia, China, and the Philippines). For the panel case, the panel URTs by Refs. [13,14], and [22], show that the series are non-stationary, and the Pedroni tests show that they are also cointegrated. Regarding causality, the results with individual data are mixed, while the results with PD show causality from GDP to EC in the SR.

#### 1.1.3 Feedback hypothesis (FH)

Güler et al. [40] analyze the shock of the Covid-19 epidemic on the ratio of EC to GDP of 30 European countries from 2015Q1 to 2021Q3. They use quarterly series of GDP and EC. The tests by Refs. [15,16] show that the variables are stationary. The causality test by Ref. [19] shows evidence in favor of the FH between EC and GDP.

In [3] it is estimated the impact of renewable EC on EG for 10 industrialized countries between 1990 and 2015. The variables used are real GDP, EC from renewable and non-renewable sources, capital formation, total work force, and an average economic openness variable. The methods used are those of [15,22] for panel URTs and those of Pedroni [28,29] for the cointegration analysis. The FMOLS technique [41] and the VECM are used to verify the DOC. As a result, the URTs show series I(1), and test by Ref. [27] presents cointegration between the variables. Moreover, a raise in EC from renewable sources has a greater effect on EG than an increase of the same magnitude in EC from non-renewable sources. Finally, the Granger test finds a bidirectional CR in the short and LR between EC from renewable sources and EG.

In [26] it is examined the causality among GDPpc, ECpc, and urbanization level in China between 2000 and 2017. In both short and LR, as the second set of results, they find evidence of a feedback CR between GDPpc and ECpc. For the full panel at the national level, the western region, and the central region FH is confirmed in the SR. Similarly, for the full panel and the western region, bidirectional CR among GDPpc and ECpc is verified in the LR.

In [42] it is analyzed the linkage between ECpc, Internet demand, and GDPpc in 35 OECD countries among 1993 and 2014, using annual data. The URTs ([15,22,32,43,44]) indicate that all variables are I(1). To capture potential LR relationships and causality, Pedroni [28,29] and Kao [45] PC tests, FMOLS, DOLS, and Dumitrescu and Hurlin tests [19] was conducted, respectively. Their results for the FMOLS and DOLS models indicate a positive association among ECpc, Internet demand, and LR EG. Dumitrescu and Hurlin's study [19] results confirm a feedback CR between ECpc and GDPpc, a causality from Internet demand to ECpc, and a causality running Internet demand toward GDPpc.

Using PD for 210 countries between 1960 and 2014, Sarwar et al. [20] analyze the nexus among GDP, EC, gross FCF, oil prices, and population. In the SR, they provide new evidence of a bidirectional CR between GDP and EC for upper middle-income countries, high income countries, full panel, OECD countries, East Asia, and Pacific. In addition, the oil price results provide evidence of a bidirectional CR between GDP and EC for the full panel, lower middle-income countries, and non-OECD countries.

Osman et al. [12] examine the nexus between ECpc and GDPpc in the Gulf Cooperation Council countries between 1975 and 2012. The tests by Refs. [[46], [47], [48]] show that the variables exhibit cross-sectional dependence and heterogeneity across groups in the PDset. The URT by Ref. [16] shows that the series are I(1). The tests by Refs. [28,29,49] indicate cointegration between all variables. The following models are used by the academics to estimate the dynamic linkages of the research variables: PMGE, demeaned PMGE, augmented mean group, mean group estimator (MGE), and dynamic fixed effects (DFE). To establish the proper model and the best estimator, the researchers performed the Hausman test. The PMGE model was found to be the most efficient of the three estimators (i.e., MGE, PMGE, and DFE) and a panel VAR was used to determine the DOC, which validated the FH in these countries.

In [10] it is estimated the linkage among ECpc and GDPpc in OPEC countries over the period 1980–2011, using annual data. As a second set of results, they find a positive bidirectional SR relationship among GDPpc and ECpc.

Karanfil and Li [31], in addition to the previously reported results, they also find LR cointegration and causality among ECpc and GDPpc for the full panel, which would constitute evidence supporting the FH.

Mohammadi and Amin [7] analyze the nexus among real GDPpc and EC for 79 countries among 1971 and 2011. Furthermore, the LR bidirectional CR between real GDPpc and EC in the three groups of countries is suggested by the results obtained through EC models. Moreover, it is confirmed that the SR CR between EC and real GDPpc is bidirectional both for the full sample of countries analyzed and for low-growth countries. Finally, in the LR, the finding of a feedback CR is shown to be robust as regards the inclusion of exports, carbon emissions, urbanization, and FDI as control variables.

As a second set of findings [2], find evidence of a bidirectional CR among EG and EG in both the short and LR for the panel of high and upper middle–income countries by applying the GC in an EC model to 88 countries grouped in four panels according to their income level between 1990 and 2006. Finally, they report a bidirectional CR in the LR for the panel of lower middle–income countries.

As a final result, Narayan et al. [34], after applying the causality test by Ref. [35] in 7 panels (Asia, Western Europe, Middle East, Africa, G6, Latin America, and global) for the period 1980–2006, find that EC and real GDP have a bidirectional GC linkage in the LR for the entire panel except for the Middle East panel.

In [23] it is examined the causality between EC, GDP and exports, for a panel of six Middle Eastern countries between 1974 and 2002. One of their findings is that the PGC test also confirms the FH in the LR.

In [36] it is studied the nexus among EC and GDP for 10 industrialized countries in Asia between 1971 and 2001, the PD results also verified the FH between GDP and EC in the LR.

#### 1.1.4 Neutrality hypothesis (NH)

Rahimi and Rad [50] analyze the dynamic impacts of Internet use and GDPpc on ECpc using PD from eight developing countries (D-8) during 1990–2013. The authors used panel URTs ([15,22,32]), PC Kao and Pedroni tests ([29]), PMGE method, and heterogeneous panel causality test by Ref. [19]. The results of this research show that all variables are I(1). The results from the PMG estimations show that Internet use positively affects ECpc only in the LR. In both the short and LR, however, GDPpc affects ECpc. Their causality results show evidence that favors the NH between GDPpc and ECpc. Furthermore, a CR from Internet use to GDPpc, and a feedback CR between ECpc and Internet use are validated, respectively.

Sarwar et al. [20] estimate the association between GDP, EC, oil price, gross FCF, and population, with PD for 210 countries between 1960 and 2014. Their final results validate the NH in the SR for low-income countries, lower middle-income countries, the Caribbean, Latin America, South Asia, North America, and Sub-Saharan Africa groups.

Karanfil and Li [31] also find evidence supporting the NH for Sub-Saharan Africa, North America, and upper middle–income countries in the SR.

## 2 Methodology

The EE from the many studies conducted shows that the DOC between EG and EC may vary depending on the methodology, countries, and periods evaluated. The present research seeks to determine the linkage among these variables, using UR, cointegration, and GC tests applied to PD. First, the URT by Ref. [15] was used to evaluate the stationarity of the variables; afterward, the cointegration among the variables was evaluated with the Pedroni [28], Kao [45] and Westerlund [49] tests. Finally, the causality test by Ref. [51] between the variables under study was identified using an EC vector model.

### 2.1 URT on PD

To evaluate the stationarity of the variables, we used the test by Ref. [15]. This test allows to consider a heterogeneous autoregressive UR process among the different sections of the panel [15]. For its calculation, ADF regressions are estimated for each section of the panel in accordance with the specification of equation (1), and then the individual t-statistics are weighted to get the final value of the test.

(1) $$\Delta y_{it}=\rho_{i}y_{it-1}+\sum_{j=1}^{p_{i}}\;\gamma_{ij}\Delta y_{it-j}+X_{it}^{\prime }\delta +\varepsilon_{it}$$

In equation (1), i=1,…,N is for the panel sections and *t* = 1, …, T indicates the time periods, $X_{it}^{\prime }$ is the set of exogenous variables, which includes the fixed effects and the individual trends, and $\varepsilon_{it}$ is the error term.

$$H_{0}:\rho_{i}=0\;\forall i$$

$$H_{1}:\rho_{i}=0,i=1,2,\ldots ,N_{1}\wedge \rho_{i}<0,i=N_{1}+1,N_{1}+2,\ldots ,N$$

### 2.2 PD cointegration test

To analyze the presence of cointegration, we use three tests (Pedroni, Kao and Westerlund tests) with the purpose of give robustness to the results. The cointegration test by Ref. [28] is implemented according to the following specification:

(2) $$y_{it}=\alpha_{i}+\delta_{i}t+\beta_{i}X_{it}+e_{it}$$

In equation (2), $\alpha_{i}$ denotes the intercept, $\delta_{i}$ represents the trend effect, $X_{it}$ denotes the regressors, and $\beta_{i}$ are the coefficients associated with the regressors in each i section of the panel. It is also assumed that variables $y_{it}$, y, and $X_{it}$ are stationary at first difference. To test the H_0_ of no cointegration, equation (3) is estimated with the residuals $e_{it}$ of equation (2), and it is analyzed whether $\rho_{i}=1$.

(3) $$e_{it}=\rho_{i}e_{it-1}+u_{it}$$

Pedroni [28] proposes seven different statistics to evaluate PC—four are based on a common term and refer to the *Within* dimension (Panel Phillips–Perron type *ϱ*–statistics, Panel ν–statistic, Panel ADF type *t*–statistic, and Panel Phillips–Perron type *t*–statistics), while the rest are based on the *Between* dimension (Group Phillips–Perron type *ϱ*–statistics, Group ADF type *t*–statistic, and Group Phillips–Perron type *t*–statistic).

In the case of Kao [45], this test is based on [37] according to the following equation:

(4) $$y_{it}=\alpha_{i}+\beta_{i}X_{it}+e_{it}$$

In equation (4), i=1,2.,N is the number of sections in the panel and t=1,2,.,T is the number of periods. Then, the test by Ref. [37] tests the H_0_ of no cointegration by using the following regression, where ${\hat{e}}_{\text{it}}$ is the estimate of $e_{it}$ in equation (4), and evaluating if ρ=1:

(5) $$e_{it}=\rho {\hat{e}}_{\text{it}-1}+u_{\text{it}}$$

Finally, Westerlund [49] estimates four panel tests, which are based on equation (6):

(6) $$\Delta y_{it}=\delta_{i}^{\prime }\partial_{t}+\alpha_{i}y_{it-1}-\varphi_{i}^{\prime }X_{it-1}+\sum_{j=1}^{p_{i}}\;\varphi_{\text{ij}}\Delta y_{it-j}+\sum_{j=1}^{p_{i}}\;\theta_{\text{ij}}\Delta X_{it-j}+e_{it}$$

Where $\alpha_{i}$ is the EC parameter, Δ is the first difference operator, $\partial_{t}$ represents the deterministic components and $p_{i}$ is the lag order that may vary across sections i of the panel. If $\alpha_{i}$ < 0 then exist error correction, which indicates that the variables are cointegrated. If $\alpha_{i}$ = 0, there is no cointegration, so the H_0_ of no cointegration for cross sectional unit i is $H_{0}:\alpha_{i}=0$.

### 2.3 GC test on PD

Cointegration among two or more variables entails the existence of a unidirectional or bidirectional CR [51]. In this study, GC is evaluated on the basis of VECM, which in the empirical literature is employed to check whether the variables are cointegrated in CRs.

Therefore, according to [26] the GC test was performed according to the following econometric specifications:

(7) $${\Delta \ln EC}_{it}=\alpha_{it}+\sum \delta_{it}{\Delta \ln EC}_{it-1}+\sum \beta_{it}{\Delta \ln EG}_{it-1}+\theta_{it}{ECT}_{it-1}+e_{1it}$$

(8) $${\Delta \ln EG}_{it}=\alpha_{it}+\sum \delta_{it}{\Delta \ln EG}_{it-1}+\sum \beta_{it}{\Delta \ln EC}_{it-1}+\theta_{it}{ECT}_{it-1}+e_{2it}$$

In equations (7), (8), ${ECT}_{it-1}$ represent the lagged EC term and, $e_{it}$ is the error. SR causality impacts are detected by the SS of the lagged explanatory variable of VECM. Similarly, LR causality between variables is identified through the SS of the lagged EC term.

The SS for both types of causality is evaluated by the Z statistic, whose value and statistical probability are reported in the estimates of both equations obtained by Stata using the PMG estimator [52]. Thus, if $\beta_{it}$ y $\theta_{it}$ are SST, there is corroborate of causality from one variable to another in the short and LR or in one of these directions.

### 2.4 The FMOLS and PMG PD models

#### 2.4.1 Theoretical model

In equation (9), a conventional model is proposed where $y_{it}$ is the explained variable and $X_{it}$ the explanatory variable:

(9) $$y_{it}=f\left(X_{it}\right)$$

Depending on the CR identified through the Granger test, the following specifications or both could be derived at a theoretical level:

(10) $$\begin{array}{l} {EC}_{it}=f\left({EG}_{it}\right)\;\\ \;\;\;\;\;\;\;\;\;\;\;\;\;\left(+\right)\; \end{array}$$

(11) $$\begin{array}{l} {EG}_{it}=f\left({CE}_{it}\right)\;\\ \;\;\;\;\;\;\;\;\;\;\;\;\;\left(+\right)\; \end{array}$$

Equation (10) reflects the energy CH, while equation (11) reflects the GH. For both relationships the relationship is direct. Thus, under the CH, it is evident that the greater the EG, the greater the EC; while under the GH, greater EC is due to greater CE.

#### 2.4.2 Econometric model

In this research, using the FMOLS model, the LR relationship between the EC and EG variables is studied, according to the DOC identified through the Granger test. This model in turn allows us to derive the LR elasticities of either EG with respect to EC, vice versa or both, if applicable. In equation (12), the FMOLS model is specified as follows:

(12) $$y_{it}=\alpha_{i}+\beta X_{it}+e_{it}$$

According to Mensah et al. [53], by estimating the FMOLS model, endogeneity bias is prevented, as well as serial correlation problems and simultaneous bias are also eliminated. In general, in this equation $y_{it}$ is defined as the explained variable and $X_{it}$ as the explanatory variable. Depending on the CR identified between both variables through the GC test, EC and EG will be specified within the equation as dependent and/or explanatory according to equations (13) and (14):

(13) $$\ln\;{EC}_{it}=\alpha_{1i}+\beta_{1}\ln\;{EG}_{it}+e_{1it}$$

(14) $${lnEG}_{it}=\alpha_{2i}+\beta_{2}\ln{EC}_{it}+e_{2it}$$

Similar treatment to the model FMOLS is given for the variables EC and EG in terms of $y_{it}$ or $X_{it}$ is provided for the specification of the SR relationship which is estimated through a VEC model in PD, its representation being through equation (15):

(15) $${\Delta y}_{it}=\beta \Delta X_{it}+\theta {ECT}_{it-1}+v_{it}$$

Being the econometrics representations for both EC and EG according to the CR resulting from the Granger test are shown in equations (16) and (17):

(16) $$\Delta \ln\;{EC}_{it}=\alpha_{1i}+\beta_{1}\Delta \ln\;{EG}_{it}+\theta_{1}{ECT}_{1it-1}+v_{1it}$$

(17) $$\Delta {lnEG}_{it}=\alpha_{2i}+\beta_{2}{\Delta \ln EC}_{it}+\theta_{2}{ECT}_{2it-1}+v_{2it}$$

Where, ${ECT}_{it-1}=y_{it-1}-\alpha_{i}-\beta X_{it-1}$ represents the correction term of the error resulting from the LR equation, that is, from the FMOLS model. For its part, $v_{it}$ it is the random error term associated with the PMG model. As indicated in Wang et al. [26] in the PMG model the LR estimators are the same, while the SR estimators (the speed of adjustment and the error variances) differ between the groups. Likewise, the SR dynamics and the LR adjustment processes are estimated simultaneously. Depending on the CR identified between EC and EG the results of this model allow us to obtain the SR elasticities of either EG with respect to EC, vice versa or both, if applicable.

### 2.5 Variables and sources of information

In this study, the EG was measured through the GDP (constant 2015 USD) per capita for each of the 31 LAC countries under study for the period 1980–2021. The data corresponding to this indicator was extracted from the World Development Indicators website.

As regards EC, it was measured through the indicator of electricity net consumption per capita (kWh per capita) for each of the 31 countries of LAC under analysis for the period 1980–2021. This indicator was calculated for each of the countries under study as the quotient of electricity net consumption (converted from billion kWh to kWh) and total population (inhabitants). The statistical information corresponding to electricity net consumption (billion kWh) was attained from the U.S. Energy Information Administration website. Statistical information on total population was obtained from the World Development Indicators website.

## 3 Results

### 3.1 URT on PD

Using the test by Ref. [15], the natural logarithm of the variables EC and GDPpc was evaluated both in level and in first difference. Similarly, the test was applied with trend and intercept and solely intercept. Table 1 displays the outcomes of the test and its variants; it is followed that, in general, the H_0_ of UR at 5 % SS cannot be rejected when the variables are evaluated in levels, but this hypothesis is rejected when the variables are evaluated in first difference. Therefore, we can say that the variables are I(1).

**Table 1:** Im–Pesaran–Shin URT.

IPS test	Statistic/Probability	lnEC	lnGDP
level (None)*	Statistic	−1.898	1.569
	Probability	0.029	0.942
1st. diff (None)*	Statistic	−20.848	−18.193
	Probability	0.000	0.000
level (Constant)	Statistic	2.005	5.676
	Probability	0.978	1.000
1st. diff (Constant)	Statistic	−20.290	−17.010
	Probability	0.000	0.000
level (C&T)**	Statistic	−2.953	−2.554
	Probability	0.002	0.005
1st. diff (C&T)**	Statistic	−20.848	−18.193
	Probability	0.000	0.000

(*) Without constant or trend (None). (**) With constant and trend (C&T).

### 3.2 Cointegration test on PD

Posterior to assessing the presence of a UR, we proceeded to evaluate the cointegration among the variables using the test by Ref. [28]. Table 2 displays the outcomes that reveal the rejection of the H_0_ of no cointegration at 1 % SS in 5 of the 7 statistics calculated. Then, it can be deduced that there is a LR relationship between EG and EC.

**Table 2:** Panel residual cointegration test. *

Test name	PC test statistics	Statistic	p_value
Pedroni	Panel ν–statistic	−1.21	0.114
	Panel ϱ–statistic	−2.60	0.005**
	Panel Pillips–Perron–statistic	−4.35	0.000**
	Panel ADF–statistic	−3.56	0.000**
	Group ρ	−1.25	0.106
	Group Pillips–Perron	−3.91	0.000**
	Group ADF	−2.92	0.002**
Kao	Augmented Dickey-Fuller	−3.26	0.001**
Westerlund	Group mean statistic $G_{\tau }$	−5.88	0.000**
	Group mean statistic $G_{\alpha }$	−5.75	0.000**
	Panel statistic $P_{\tau }$	−2.27	0.012***
	Panel statistic $P_{\alpha }$	−3.15	0.000**

Developed by authors. *A constant and linear trend are considered in the cointegration relations. ** p<0.01.***p<0.05.

### 3.3 GC test on PD

As per Table 3, the VECM GC reveals two main results. First, EG (GDP) causes EC in both the short and LR. This result depends of the SS of ${\Delta lnGDP}_{i,t-1}$ and ${ECT}_{i,t-1}$ when considering ${\Delta lnEC}_{i,t}$ as the dependent variable**.** Second, it is confirmed that EC causes GDP only in the LR. This result is validated through the SS of ${ECT}_{i,t-1}$ when a ${\Delta lnGDP}_{i,t}$ is considered as the dependent variable. In this sense, considering both results, we conclude that EG causes EC in the SR, while the causality between both variables is bidirectional in the LR.

**Table 3:** Panel VECM GC test.

Types of Causalities	Lagged Variables	Dependent Variables	
		${\Delta \text{lnEC}}_{i,t}$	${\Delta \text{lnGDP}}_{i,t}$
SR causality	${\Delta \text{lnEC}}_{i,t-1}$	−0.0344	−0.0052
		(0.0425)	(0.0188)
	${\Delta \text{lnGDP}}_{i,t-1}$	0.1940*	0.1968**
		(0.0939)	(0.0371)
LR causality	${\text{ECT}}_{i,t-1}$	−0.1089**	−0.1058**
		(0.0212)	(0.0220)

Developed by authors. *p<0.05.**p<0.01. Note: The standard deviations of the estimators obtained are expressed in parentheses. The estimated models for ${\Delta \text{lnEC}}_{i,t}$ and ${\Delta \text{lnGDP}}_{i,t}$ incorporate the intercept; however, Table 3 does not include this estimator since its interpretation is not relevant for the purpose of analyzing the results.

### 3.4 The long- and SR relationship between EC and EG

Table 4 displays the outcomes of the short- and LR relationships among EC and EG. The identified relationships stand out for their 1 % level of SS, which demonstrates that each of the variables is highly important to explicate the behavior of the other. These results, in turn, are discussed in detail in the following section with emphasis on the elasticities obtained among both variables under study in both the short and LR.

**Table 4:** Panel estimates between EC and EG.

	${\text{lnEC}}_{i,t}$	${\text{lnGDP}}_{i,t}$
FMOLS: LR estimates		
${\text{lnEC}}_{i,t}$	–	0.4971**
	–	(0.0151)
${\text{lnGDP}}_{i,t}$	1.5443**	–
	(0.0468)	–
PMG: SR estimates		
${\text{ECT}}_{i,t-1}$	−0.1150**	–
	(0.0176)	–
${\Delta \text{lnEC}}_{i,t}$	–	–
	–	–
${\Delta \text{lnGDP}}_{i,t}$	0.5320**	–
	(0.0994)	–

Developed by authors. ** p<0.01. Note: The standard deviations of the estimators obtained are expressed in parentheses. The FMOLS model incorporate the intercept; however, Table 4 does not include this estimator since its interpretation is not relevant for the purpose of analyzing the results. For its part, the PMG model does not include intercept since its estimation does not require said parameter as shown in section 2.4.

## 4 Discussion

Based on our results, we validate a CR in the SR from GDP to EC. Thus, in the SR, there is evidence supporting the energy CH which, in Ozturk's words (see Ref. [4]), implies that electricity CP can be executed with little or no negative shock on EG. Therefore, LAC countries are characterized in the SR as economies that are not dependent on electricity. Nevertheless, in the LR, there is evidence of a bidirectional RC among EC and GDP, supporting the FH which indicates that GDP and EC are mutually defined and impacted concurrently.

Similar to our study, Chen et al.’s study [36] involving 10 developed Asian countries, Karanfil and Li's [31] involving 160 countries, Apergis and Payne’s [2] involving high and medium–income countries, Mohammadi et al.’s [7] involving 79 countries with different GR, Azam et al.’s [3] involving 10 developed countries in the Americas, Asia, Europe, and Africa, and Wang et al.’s [26] involving the western and eastern regions of China and China as a whole reported evidence that supports the CH in the SR and the FH in the LR.

In contrast [23], for six Middle Eastern countries and [34] for a panel of six countries (Latin America, Asia, Western Europe Africa, Middle East, and the world) verify the FH in the LR. Equally, Sarwar et al. [20] for 210 countries and Abdoli [10] for OPEC countries validate the CH in the SR.

Finally [12], for GCC countries [42], for 35 OECD countries, and [40] for 30 European countries find evidence that supports the FH among a group of articles that empirically examine the CR between EG and EC, without making a difference pertaining to the short and LR. Similarly [13], obtain results supporting the CH and FH for a set of Indian states.

Thus, based on the above results, for the 31 LAC countries analyzed, it is concluded that EC as an indicator of development can be boosted in the SR by EG. In other words, more people can demand more electricity given the income increase they may have due to greater EG. However, it is recommended to consider that EC also has an impact on EG in the LR; thus, adverse situations can limit EG in energy-dependent economies.

In this sense, although EG can improve the infrastructure (access) to electric energy in the LR, the results obtained also allow us to infer that the transmission channel of electric energy to EG in the LR should not be neglected, as the improper use of this energy source can lead to environmental degradation due to greater GHG emissions. In conclusion, the results obtained allow us to reflect on the importance of economic and environmental balance, which is necessary to maintain SD, that is, to guarantee the well-being of present and future generations.

### 4.1 A tradeoff between economy and environment

Although the results of the cointegration and GC relationship have allowed us to corroborate the existence in favor of the LR FH between EC and EG as well as the SR CH, it should be noted that in comparison to Sarwar et al. [20], the results obtained in our research show a tradeoff between economy and environment, since the aforementioned authors, although within the EE they explore the link between EC and EG, their findings for LAC reveal evidence of neutrality in both variables in both the short as well as the LR.

On the other hand, although Karanfil and Li [31] also analyze the relationship between EC and EG in LAC, their results show evidence in favor of the GH and CH in the SR, while in the LR there is evidence only the CR that goes from EG to EC, which is equivalent to the CH in the LR and therefore does not allow verifying evidence in favor of the FH in the LR between EC and EG.

However, it should be noted that given the nature of the econometric specification of the model (in levels) used by Ref. [31], it is not possible to appreciate the degree of sensitivity or elasticity in which the EC and EG variables affect each other. So then, with respect to Sarwar et al. [20], the present research shows evidence that in LAC the variables EC and EG are not independent of each other. While with respect to Karanfil and Li [31], the present research allows identifying the degree of sensitivity (elasticity) of one variable with respect to another. In this sense, the results obtained here allow us to verify the existence of a tradeoff between economy and environment. Thus, according to Tables 4 and it is observed that in the SR, electrical energy is a necessary good since its income elasticity is 0.5, that is, for every 1 % increase in GDPpc, the ECpc increases by 0.5 %.

Nevertheless, in the LR the situation is different, revealing that electrical energy is a luxury good, since its income elasticity is greater than one, in particular its value is 1.54, which reveals that for every 1 % increase In GDPpc, ECpc grows by 1.54 %, thus the raise in ECpc is greater than the magnitude of the change in GDPpc. This last finding strengthens the importance of electrical energy for the environment, since, if faced with a change in GDPpc, the level of ECpc increases by a greater magnitude than the change registered in GDPpc, this would lead to a faster GR in the flow of CO_2_ emissions, this being of special interest in a context in which at a global level it is seen that the electricity sector is the one with the highest CO_2_ emissions, while in LAC it is the third sector (of a total of eight) of higher CO_2_ emissions [6].

The result associated with electric energy as a luxury good in the LR is also reported in Rahimi and Rad [50] for a panel of 8 developing countries. Instead, the finding of electrical energy as a necessary good in the SR reported in the present research shows correspondence with Wang et al. [26] for the provinces of China and Rahimi and Rad [50]. Finally, with respect to the CR from GDPpc to ECpc in the LR, according to Tables 4 and it is observed that GDPpc responds to a lesser magnitude to the increase in ECpc, in particular, for each increase of 1 % in ECpc, GDPpc increases by 0.5 %.This finding shows correspondence with what was reported by Ref. [8] for Australia, Sarwar et al. [20] although with exception for LAC [10], for OPEC countries [7], for 79 countries [2], for 88 countries and [23] for 6 Middle Eastern countries. However, although the value of the elasticity of the EG with respect to the EC reflects an inelastic character, that is, the EG is not very sensitive to changes in EC, this does not allow us to rule out the importance of electrical infrastructure for EG in the LR, given that the Granger test shows that EG depends on EC.

The latter, in turn, does not allow us to fail to demonstrate the existence of a tradeoff between economy and environment, since the EC also depends on the EG. Thus, as EG increases, EC also increases, leading to an increase in environmental degradation through greater CO_2_ emissions. Additionally, according to Ozturk [4], although the GH implies that energy is constituted as a relevant production factor for the economy, the occurrence of negative shocks in the energy supply would have a negative effect on the EG. Therefore, achieving balanced SD in LAC in terms of economy and environment implies balancing this tradeoff through policies that promote EG based on prudent use of electric energy infrastructure. In this regard, the following section presents some proposals that could lead to balancing the tradeoff in question.

## 5 Conclusions

As the main conclusions of the research, first of all, for the period 1980–2021, the linkage among EC and EG for 31 countries in LAC is bidirectional in the LR, while in the SR, the CH is confirmed, that is, EG causes EC. Second, electricity in LAC in the SR is a necessary good while in the LR it can become a luxury good. Last, the bidirectionality of the LR relationship between EC and EG, as the luxury good nature of this first variable implies a tradeoff between economy and environment, since although greater electrical infrastructure drives EG, the latter also increases the EC whose use in a non-responsible manner can lead to environmental degradation through higher CO_2_ emissions.

These results have important consequences for economic policy. First, the investment in infrastructure (electricity) is an important element for higher levels of EG, in the LR. Second, access of electrical energy allows to achieve positive results at a social level, reflected in better education, health, work and productivity. And third, LAC EG cannot be separated from the environment, as achieving harmonious SD requires attaining a balance between economic, social, environmental, and institutional aspects. Both infrastructure and the environment are determining factors for better development opportunities for the population. Therefore, it is necessary to promote EG based on infrastructure focused on SD, ensuring the well-being of present and future generations. The SD goals in force since 2015 point toward countries with better economic, social, environmental, and institutional opportunities. Therefore, an optimal interaction between economic, social and environmental aspects is key for countries of LAC to achieve true SD, as is expected by 2030.

On the other hand, with respect to future research, it should be previously noted that, first of all, the vast majority of empirical studies of the nexus among energy and EG are characterized by generally incorporating energy consumption at an aggregate level into their analysis. Secondly, although there is EE that documents the link between electric energy (without considering other types of energy) and EG, for LAC only two studies have been identified that incorporate this region as a study sample but not as a particular case of analysis such as that of the present investigation.

Thus [20], concluded that there is no causality among the research variables, while that in Ref. [31] although causality is identified from EG to EC in the LR and from EC to EG in the SR, it is not possible to determine in terms of elasticity the magnitude of impact of one variable with respect to another given the econometric specification in levels used by said authors.

Therefore, this research allows us to display a tradeoff between economy and environment, that is, the relationship between EC and EG goes beyond causality since in a context of SD, in addition to greater access to electric energy as inclusive growth, the environmental dimension is relevant through the reduction of CO_2_ emissions.

Hence, the objective of this research having been a new empirical approach between EC and EG, for future research in order to validate the robustness of the relationship obtained, it is recommended to include control variables such as the price of electric energy, FDI, urbanization or others in depending on the statistical information available.

On the other hand, although this research mentions that the effect of greater EC translates into an increase in CO_2_ emissions, this is based on a conceptual level as well as the available statistics that show that greater CO_2_ emissions come from the electricity industry both in the world and in LAC (third generating sector). In this sense, as a future research perspective, it is considered pertinent to carry out a study that quantitatively confirms the nexus among EC, EG and CO_2_ emissions.

Finally, another possible future line of research would consist of analyzing in LAC the relationship between EG and energy consumption (not only electrical consumption) considering other control variables (trade openness, labor, industrialization, domestic financial assistance, population, among others) as well as identifying its contribution to CO_2_ emissions, as has been carried out in research that considers other regions of the planet: [[54], [55], [56], [57], [58]].

## Data availability statement

The data used in this paper has not been deposited into a publicly available repository and it will be available from the corresponding author upon request.

**Declaration of competing interest:** The authors declare that they have no known competing financial interests or personal relationships that could have appeared to influence the work reported in this paper.

## Ethics statement

The authors of this paper declare their commitment and adherence to all the provisions of the ethical statement of this Journal. The authors want to make it clear that they didn't use human or animal samples in their study, so they didn't have required a special authorization. Likewise, the authors want to reported that this Journal has permission to publish this paper.

## CRediT authorship contribution statement

**Ciro Eduardo Bazán Navarro:** Writing – review & editing, Writing – original draft, Supervision, Project administration, Conceptualization. **Juan Daniel Morocho Ruiz:** Writing – review & editing, Writing – original draft, Methodology, Data curation, Conceptualization. **Juan Francisco Castillo Alvarado:** Writing – review & editing, Writing – original draft, Methodology, Data curation, Conceptualization.

## Declaration of competing interest

The authors declare that they have no known competing financial interests or personal relationships that could have appeared to influence the work reported in this paper.
