# Supplementary material for: Economic growth and electricity consumption: Fresh evidence of panel data for LAC
Source: Heliyon. 2024 Jun 25;10(13):e33521. doi: 10.1016/j.heliyon.2024.e33521 (PMC11261010; doi:10.1016/j.heliyon.2024.e33521)
Supplement: Multimedia component 1 [file mmc1.docx]

**Table A.** Empirical literature on electricity consumption-economic growth relationship.

| **Authors** | **Period** | **Country** | **Methodology *** | **Results **** |
| --- | --- | --- | --- | --- |
| ***Bivariate Models*** |  |  |  |  |
| Osman et al. (2016) | 1975-2012 | The GCC countries | - Tests of cross-sectional dependence: Breusch and Pagan (1980), Pesaran (2004) and Baltagi et al. (2012). - Unit root tests: Pesaran (2007). - Panel cointegration test: Pedroni (1999) and Westerlund (2007). - PMGE, DPMG, AMG, MGE, and DFE models. - Hausman`s test. - Panel VAR Granger Causality test. | - GDP an EC are I(1). - GDP and EC are cointegrated. - Granger Causality:   Short-term: GDP↔EC  Long-term: GDP↔EC |
| Karanfil and Li (2015) | 1980–2010 | 160 countries (full panel, OECD membership, income level, region) | - Unit root tests: Levin et al. (2002), Im et al. (2003). - Granger Causality is contrasted with a VEC model (for cointegrated panels) and with a VAR model (for non-cointegrated panels). - Panel cointegration tests: (Pedroni, 1999, 2004). | - GDP and EC are I(1) - GDP and EC are cointegrated. - Granger Causality:   Short-term: GDP→EC (East Asia and the Pacific, the Middle East and North Africa and in lower-middle-income countries). GDP−EC (North America, Sub-Saharan Africa and upper-middle income countries). |
| Narayan et al. (2010) | 1980- 2006 | 07 panels: Western Europe, Asia, Africa, Middle East, Latin America (17 countries), G6, and global | - Unit root test: Pesaran (2007). - Panel cointegration: Pedroni (1999, 2004) - Panel Causality test: Canning and Pedroni (2008). | - GDP and EC are I(1) for all panels - GDP and EC are cointegrated for all seven panels. - Granger causality (using LP test because is most realiable): - Long term: EC → GDP (For all panels except for the Middle East. For the G6 panel, causality is negative) GDP → EC All panels |

| **Table A. (continued).** | | | | | | |  |
| --- | --- | --- | --- | --- | --- | --- | --- |
| **Authors** | **Period** | **Country** | | **Methodology *** | **Results **** | |  |
| ***Bivariate Models*** |  |  | |  |  | |  |
| Chen et al. (2007) | 1971-2001 | 10 newly industrializing and developing countries Asian countries | | - Unit root test: Dickey and Fuller (1981), Phillips and Perron (1988), Im et al. (2003), Levin et al. (2002), and Hadri (2000). - Causality test: Yoo (2005). - Granger Causality is contrasted with the ECM estimated by the 2-stage procedure of the Engle and Granger (1987) model. using a VECM model - Cointegration tests: Johansen and Juselius (1990), and Pedroni (1999, 2004). | - GDP and EC are I(1). - GDP and EC are cointegrated. - Granger causality in panel data:   Short-term: GDP → EC  Long-term: GDP↔EC | |  |
| ***Multivariate Models*** | | | | | | |  |
| Güler et al. (2022) | 2015Q1-2021Q3 | 30 European countries | - Test of cross-sectional dependence: Pesaran (2004). - Unit root tests: Im et al. (2003), Pesaran (2007). - Homogeneity tests: Swamy (1970) and Pesaran and Yamagata (2008). - Heterogeneous panel causality test: Dumitrescu and Hurlin (2012). - Generalized moment method (GMM) dynamic panel estimation. | | | - GDP and EC are I(0) - There is bidirectional causality: GDP↔EC. - The negative impact of EC on GDP during strict confinement period (2015Q1–2020Q3) of the COVID-19 pandemic is supported. | |
| Azam et al. (2021) | 1990-2015 | Mexico, Brazil, Turkey, Thailand, South Africa, Philippines, Malaysia, Indonesia, India and China | - Unit root tests: Levin et al. (2002) and Im et al. (2003). - Panel cointegration tests: Pedroni (1999, 2004). - FMOLS: Pedroni (2000). - Panel VECM Granger causality. | | | - GDP, REC, NREC, GKF, LF are I(1) - GDP, REC, NREC, GKF, LF and TO are cointegrated. - A 1% increase in REC will increase GDP by 0.095% and GDP increases by 0.017% if NREC increases by 1%. - Granger Causality:   Long term: REC↔GDP NREC↔GDP  Short term: REC↔GDP NREC→GDP | |

**Table A.** (continued).

| **Authors** | **Period** | **Country** | **Methodology *** | | | **Results **** |  |
| --- | --- | --- | --- | --- | --- | --- | --- |
| ***Multivariate Models*** | | | | | | |  |
| Wang et al. (2021) | 2000-2017 | China (at the national and regional levels) | - Unit root tests: Im et al. (2003). - Panel cointegration test: Pedroni (2001). - Panel VECM Granger causality. - PMG estimator. | | | - All variables are I(1) - GDP, EC and UI are cointegrated - Granger causality:   Short-term: GDP↔EC, UI↔GDP and UI$↛$EC (At the national level), GDP→EC, GDP→UI (Eastern region), EC↔GDP, EC→UI, (Central region), GDP↔EC, UI→GDP and UI$↛$EC (Western region).  Long-term Granger causality: GDP↔EC, UI↔GDP and UI↔EC (At the national level and Eastern region), GDP→EC (Central region), GDP↔EC (Western region), UI$↛$GDP and UI$↛$EC (Central and western regions). |  |
| Tiwari et al. (2021) | 1960/61 - 2014/15 | India (18 States) | - Cross-sectional dependence test: Pesaran (2004). - Unit root tests: Pesaran (2007), Im et al. (2003), Hadri (2000) and Nazlioglu and Karul (2017). - Cointegration test: Westerlund(2007) and Westerlund and Edgerton (2008) - Heterogeneous panel causality test: Dumitrescu and Hurlin (2012). - Impulse-response function in a panel VAR model. | | | - All variables are I(1) - NSDPA and EC are cointegrated (agricultural sector). - Granger causality:   EC→ NSDPA (agricultural sector).  NSDP→EC (aggregate state level).  NSDPI→EC (industrial sector). |  |
| Churchill and Ivanovski (2020) | 1990-2015 | 7 Australian states/territories | - Tests of cross-sectional dependence: Pesaran (2004). - Cross-sectionally ADF unit root test: Pesaran (2007). - Panel cointegration tests: Pedroni (1999, 2004), Kao (1999), and Fisher-Johansen. - Panel elasticities estimations: DOLS, FMOLS, and ARDL models. - Heterogeneous panel causality test: Dumitrescu and Hurlin (2012). | | | - Variables are I(1) - GSP, EC, GKF, and LF are cointegrated - The long-term and short-term results suggest that EC increase GSP, but some differences are observed across the states and territories between EC and GSP. - Causality direction:   EC→GSP, GSP→LF, EC→GKF, GKF↔GSP, LF↔EC, LF↔GKF. |  |
| **Table 1.** (continued). | | | | | | | |
| **Authors** | **Period** | **Country** | | **Methodology *** | **Results **** | | |
| ***Multivariate Models*** | | | | | | | |
| Khobai (2018) | 1990-2014 | Brazil, Russia, India, China and South Africa (BRICS countries) | | - Unit root test: Im et al. (2003), ADF-Fisher Chi-square and PP-Fisher Chi-square . - Panel cointegration tests: Kao (1999) and Johansen-Fisher. - Panel Granger causality test based on VECM. | - Cointegration: there is a long-term relationship between the EC, GDP, carbon dioxide emissions and urbanization. - Long-term Granger causality tests: the causality goes from GDP, CO_2_ and urbanization to the EC, and EC$↛$GDP. - Short-term Granger causality tests: EC→ GDP. | | |
| Kirikkaleli et al. (2018) | 1993-2014 | 35 OECD countries | | - Unit root tests: Maddala and Wu (1999), Choi (2001), Levin et al. (2002), Im et al. (2003), and Breitung and Candelon (2005). - Panel cointegration tests: Pedroni, (1999), and Kao (1999). - Panel FMOLS and DOLS estimators. - Heterogeneous panel causality test: Dumitrescu and Hurlin (2012). | - The cointegration tests and the results of the FMOLS and DOLS estimators confirm a positive cointegration relationship between EC, ID, and long-term economic growth. - Dumitrescu-Hurlin panel causality test: GDP↔EC (at the 1% significance level), ID→EC (at the 10% significance level), and ID→GDP (at the 5 % significance level). | | |
| Rahimi and Rad (2017) | 1990-2013 | Countries of the Organization for Economic Cooperation (D-8) | | - Unit root tests: Maddala and Wu (1999), Levin et al. (2002), and Im et al. (2003). - Panel cointegration tests: Pedroni (2004), and Kao (1999). - PMG estimations. - Heterogeneous panel causality test: Dumitrescu and Hurlin (2012). | - Evidence of cointegration between EC and GDP in the long-term. - Long-term effect of the UI on the EC: A 1% increase in the number of Internet users per 100 people increases the EC per capita by 0.036%. - Short and long-term effects of the GDP on the EC: An economic growth rate of 1% will cause an increase of 1.11% in the EC per capita. - Granger causality: EC–GDP, UI↔EC, UI→GDP, GDP$↛$UI. | | |

| **Table 1.** (continued). | | | | |
| --- | --- | --- | --- | --- |
| **Authors** | **Period** | **Country** | **Methodology *** | **Results **** |
| ***Multivariate Models*** | | | | |
| Sarwar et al. (2017) | 1960-2014 | 210 countries (full panel, income level, OECD, region, renewable energy, oil import/export) | - Panel unit root test: ADF and PP Maddala and Wu (1999), and Levin et al. (2002). - Panel cointegration tests: Pedroni (1999, 2004, 2000, 2001). - FMOLS estimator. - Panel Granger causality test based on VECM. | - Short-term Granger causality tests: The FH (full panel, upper-middle income, high income, OECD, EAP), the GH (NOECD, ECA and MENA), and the NH (low income, low-middle income, LAC, NA, SA and SSA region) are verified between EC and GDP. The results of oil price present: EC↔GDP (full panel, low-middle income and NOECD countries); OP→GDP (low income, EAP and NA); GDP→OP (upper-middle income, high income, OECD, ECA, SA and SSA region); and OP–GDP (LAC, MENA region). - Long-term dynamic: exist a significant negative relationship between EC and GDP (low income, upper-middle income, high income, OECD, EAP, ECA, SA, full panel and MENA). There is a significant negative relationship between OP and GDP (full panel, upper-middle income, high income, OECD, EAP, LAC and SSA region), and exist a significant positive relationship between OP and GDP (low-middle income, NOECD, MENA and SA). Exist a significant positive relationship between GKF and GDP (full panel, low-middle income, upper-middle income, ECA, MENA, NA and SSA countries). |
| Raza et al. (2016) | 1980-2010 | South Asian countries: Pakistan, India, Bangladesh and Sri Lanka | - Unit root tests: Im et al. (2003). - Panel cointegration test: Pedroni (1999). - Wald and Hausman Tests. - Panel POLS, GMM, FMOLS and DOLS estimators. - Panel Granger causality test. | - There exists long-run relationship between EC and GDP. - Estimations of REM suggest a positive and significant impact of EC on GDP. - Granger causality: EC→GDP. |

| **Table 1.** (continued). | | | | | | | |  |
| --- | --- | --- | --- | --- | --- | --- | --- | --- |
| **Authors** | **Period** | **Country** | | **Methodology *** | | **Results **** | |  |
| ***Multivariate Models*** | | | | | | | |  |
| Abdoli et al. (2015) | 1980-2011 | OPEC countries | | - Unit root tests: Im et al. (1997). - Panel cointegration tests: Pedroni (1999, 2004). - FMOLS estimator. - Panel Granger causality test | | - Cointegration between GDP, EC and commercial activities (includes exports and imports). - Short-term Granger causality tests: GDP ↔EC. - Long-term Granger causality tests: GDP→EC, but not in the opposite direction. | |  |
| Mohammadi and Amin (2015) | 1971-2011 | 79 countries with different growth rates: 14 high-growth countries, 55 low-growth countries and 10 negative-growth countries | | - Unit root tests: Maddala and Wu (1999) and Pesaran (2007). - Panel cointegration tests: Pesaran (2006). - Causality is contrasted with ECM using the CCEMG estimator. | | - Cointegration between energy (electricity) consumption and GDP in high- and low-growth panels is verified, but absence of cointegration is reported in the panel with negative growth. - Estimates of long-term elasticities of GDP with respect to the EC are significant in panels with positive growth rates. - The mean-group estimators of ECM suggest that in the long run: GDP ↔ energy (electricity) consumption in the 3 groups of countries. In the long-term two-way causality is robust to the inclusion of carbon emissions, urbanization, exports, and FDI as control variables. In the short-term, GDP↔EC for the complete sample and for low-growth countries and GDP→ energy consumption for the negative-growth group. | |  |
| Apergis and Payne (2011) | 1990-2006 | 88 countries categorized into 4 panels (high, upper middle, lower middle, and low income) | | - Unit root tests: Levin et al. (2002) and Im et al. (2003). - Panel cointegration tests: Larsson et al. (2001). - Panel Granger causality test based on VECM. | | - There is cointegration between real GDP, coal consumption, GKF, and the LF for the panels of high, upper-middle, and lower-middle income countries. - The results of the VECM reveal: (a) bidirectional causality between EC and real GDP in both the short and long-term for the panels from high- and upper-middle-income countries; (b) for the panel of lower-middle-income countries, unidirectional causality is observed from EC to real GDP in the short term and bidirectional causality in the long- term; (c) for the panel of low-income countries, since the variables are not cointegrated, an autoregressive panel is used to determine the causal relationship, observing a unidirectional relationship from EC to real GDP. | |  |
| **Table 1.** (continued). | | | | | | | | |
| **Authors** | **Period** | | **Country** | | **Methodology *** | | **Results **** | |
| ***Multivariate Models*** | | | | | | | | |
| Ciarreta and Zarraga (2010) | 1970-2007 | | 12 European countries | | - Unit root tests: Hadri (2000), Levin et al. (2002) and Im et al. (2003). - Panel cointegration tests: Pedroni (1999). - Panel Granger causality test based on VECM. | | - The GDP, EC and EP are cointegrated series. - In the short-term: EC→GDP (negative sign relation), EP↔GDP, EC→EP. | |
| Narayan and Smyth (2009) | 1974-2002 | | 6 Middle Eastern countries | | - Unit root tests: Breitung (2000). - Panel Granger causality test. - FMOLS estimators. - Panel cointegration tests: Westerlund (2006). | | - In the short-term: EC→GDP, GDP→X. In the long-term: EC↔GDP, X→GDP, X→EC. - The FMOLS results show (cointegration): An increase of 1% in the EC increases GDP by 0.04%. An increase of 1% in X increases GDP by 0.17%. An increase of 1% in the GDP generates an increase of 0.95% in the EC. | |
| (*) ADF: Augmented Dickey-Fuller, AMG: Augmented Mean Group, CCEMG: Common correlated effect mean-group estimator, DOLS: Dynamic Ordinary Least Squares, FMOLS: Full Modified Ordinary Least Squares, PMG: Pooled Mean Group, ECM: Error correction model, MS-VAR: Markov-Swiftching VAR, MS-Granger: Markov-Swiftching Granger, OP: Oil Price, PP: Phillips-Perron, POLS: Pooled Ordinary Least Square, VAR: Vector Autoregressive, VECM: Vector Error Correction Model. (**) EAP: East Asia & Pacific, EC: Electricity consumption, ECA: Europe & Central Asia, EP: Electricity price, FDI: Foreign direct investment, FH: Feedback Hypothesis, GH: Growth Hypothesis, GSP: Gross state product, GKF: Gross capital formation, ID: Internet Demand, IU: Internet Use, LAC: Latin America & Caribbean, LF: Labor Force, MENA: Middle East & North Africa, NA: North America, NH: Neutral hypothesis, NOECD: nonOECD, NREC: Non-renewable electricity consumption, NSDP: per capita net state domestic product, NSDPA: per capita net state domestic product in the agricultural sector, NSDPI: per capita net state domestic product in the industrial sector, REC: Renewable electricity consumption, REM: Random Effects model, TO: Trade openness, UI: Urbanization indicators, SA: South Asia, SSA: Sub-Saharan Africa, X: Exports, → unidirectional causality, ↔ bidirectional causality, $↛$ indicates no causality unidirectional, and – indicates no causality in both directions. | | | | | | | | |
